# The dNTP triphosphohydrolase activity of SAMHD1 persists during S-phase when the enzyme is phosphorylated at T592

**DOI:** 10.1080/15384101.2018.1480216

**Published:** 2018-07-24

**Authors:** Elisa Tramentozzi, Paola Ferraro, Manzar Hossain, Bruce Stillman, Vera Bianchi, Giovanna Pontarin

**Affiliations:** a Department of Biology, University of Padova, Padova, Italy; b Cold Spring Harbor Laboratory, Cold Spring Harbor, NY, USA

**Keywords:** Deoxynucleotide metabolism, Sterile alpha motif and HD domain containing protein1 (SAMHD1), cell cycle

## Abstract

SAMHD1 is the major catabolic enzyme regulating the intracellular concentrations of DNA precursors (dNTPs). The S-phase kinase CDK2-cyclinA phosphorylates SAMHD1 at Thr-592. How this modification affects SAMHD1 function is highly debated. We investigated the role of endogenous SAMHD1 phosphorylation during the cell cycle. Thr-592 phosphorylation occurs first at the G1/S border and is removed during mitotic exit parallel with Thr-phosphorylations of most CDK1 targets. Differential sensitivity to the phosphatase inhibitor okadaic acid suggested different involvement of the PP1 and PP2 families dependent upon the time of the cell cycle. SAMHD1 turn-over indicates that Thr-592 phosphorylation does not cause rapid protein degradation. Furthermore, SAMHD1 influenced the size of the four dNTP pools independently of its phosphorylation. Our findings reveal that SAMHD1 is active during the entire cell cycle and performs an important regulatory role during S-phase by contributing with ribonucleotide reductase to maintain dNTP pool balance for proper DNA replication.

## Introduction

The Sterile α motif domain and histidine-aspartate domain-containing protein 1 (SAMHD1) is a nuclear triphosphohydrolase that cleaves all four deoxynucleoside triphosphates (dNTPs) to deoxynucleosides and inorganic triphosphate. The catalytic activity is regulated by nucleoside triphosphate binding at two allosteric sites, which induces the formation of a stable tetramer [1]. In addition to the well-established dNTPase activity, single stranded nucleic acid binding [2] and *in vitro* nuclease activity were reported [3,4]. However, later data attributed the nuclease activity to contaminants co-purifying with SAMHD1 and the question of SAMHD1 harboring multiple functions is still debated [5].

SAMHD1 is expressed at variable levels in most human tissues, especially in immune cells. It has been intensively investigated as a host restriction factor that, in quiescent/differentiated cells, limits HIV-1 and other viral infections by lowering cellular dNTP concentrations under a threshold critical for the synthesis of viral DNA [6].

SAMHD1 gene mutations are associated with the Aicardi-Goutières syndrome (AGS), a severe inflammatory encephalopathy characterized by inappropriate immune activation [7]. Both in AGS individuals and transgenic models the loss of SAMHD1 results in increased cellular concentrations of dNTPs [8]. SAMHD1 mutations occur in leukemias [9] and other types of human cancer, suggesting that a surplus of dNTPs contributes to cell transformation by affecting the fidelity of DNA synthesis.

SAMHD1 is a component of the enzyme network that controls dNTP levels [10]. In mammalian cells the concentrations of dNTPs are regulated with cell division cycle progression. During S-phase, the pools expand due to the induction of ribonucleotide reductase (RNR), the major anabolic enzyme providing deoxynucleotides for DNA replication. Outside S-phase, RNR activity is restricted by the ubiquitin-dependent degradation of its R2 subunit [11,12], with concomitant contraction of dNTP pools. In G1 and in quiescent cells, p53R2, the stable small subunit of RNR, provides dNTPs for DNA repair and mitochondrial DNA maintenance [13]. SAMHD1 is present during the whole cell cycle and prevents overproduction of dNTPs. Nevertheless, it is still unclear if SAMHD1 activity and protein concentration are regulated and whether SAMHD1 regulation is inversely related to that of RNR.

SAMHD1 is phosphorylated at threonine 592 (T592) by the cell-cycle regulated kinases CDK2/1 [14–16]. Phosphorylated T592 is believed to have a regulatory function but how it relates to SAMHD1 activity and/or protein stability is still questioned. Biochemical studies with recombinant phosphomimetic (T592D/E) and non-phosphorylatable (T592A/V) SAMHD1 mutants yielded conflicting results regarding tetramer stability and enzymatic properties [15,17–21]. In live cells, the effects of SAMHD1 phosphorylation were investigated by ectopic over-expression of SAMHD1 mutants and the restriction of viral infection or dNTP pool decrease, both readouts of SAMHD1 activity. In PMA differentiated U937 cells, phosphomimetic SAMHD1 mutants lacked retroviral restriction although they decreased cellular dNTP concentrations as did wild type SAMHD1 and its non-phosphorylatable mutants [15,20–22]. In proliferating cells, none of the tested SAMHD1 variants blocked retroviral infection, presumably due to the high expression of RNR that opposed the catabolic activity of SAMHD1[22]. Interestingly, only the non-phosphorylatable SAMHD1 mutants reduced the percentage of cells in S-phase and activated the DNA damage check-point[18].

No study so far has investigated SAMHD1 dephosphorylation nor looked for the protein phosphatases involved.

With this background in mind we wished to address the timing and role of SAMHD1 phosphorylation during cell cycle progression. We chose the strategy of correlating endogenous SAMHD1 phosphorylation with the dNTP levels in the individual phases of the cell division cycle, comparing parental SAMHD1-proficient and SAMHD1-KO cell lines. We investigated the regulation of SAMHD1 phosphorylation by kinase and phosphatase activities in synchronized cultures. Moreover, we tested the possibility that T592 phosphorylation acts as a signal for degradation, by measuring the turn-over of the protein in cycling cells. We suggest that SAMHD1 is a long-lived protein, active in intact cells during the entire cell division cycle independently of T592 phosphorylation, that together with RNR adjusts the dNTP pools to the requirements of DNA synthesis during S-phase.

## Results

### The absence of SAMHD1 in THP-1 KO cells leaves the expression of RNR subunits unaffected and causes a strong increase in dNTP pools in all phases of the cell cycle

**Figure 1.**
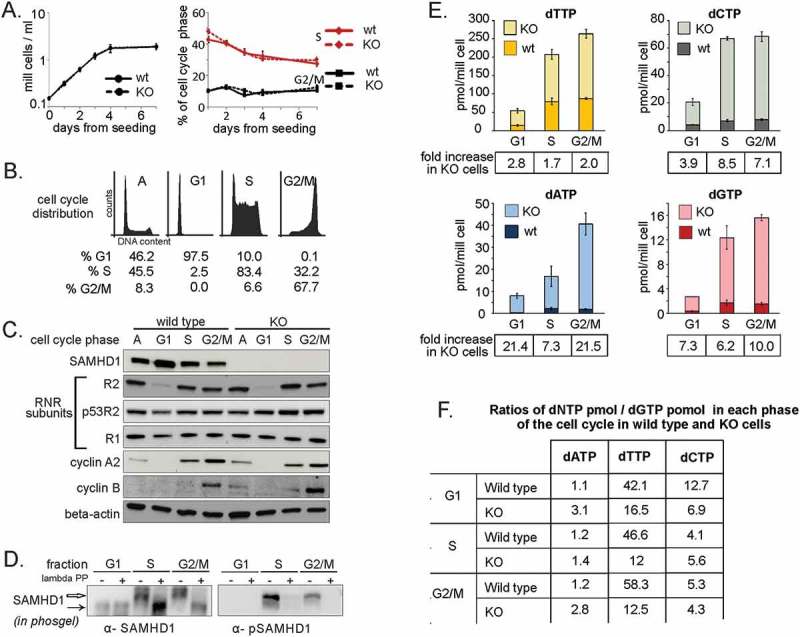
dNTP pools in different phases of the cell cycle in SAMHD1-KO and parental THP-1 cells. **A**. cell proliferation curve and cell cycle analysis of THP-1 cells knock-out for SAMHD1 (KO – dashed line) and the parental control (wt – solid line). Bars: Mean ± standard error for n = 3 values. **B**. Representative image of the cell cycle distribution of THP1 cells before (A = asynchronous population) and after centrifugal elutriation (G1, S and G2/M enriched sub-populations). **C**. Immunoblotting for SAMHD1, RNR subunits and cell cycle markers in each enriched sub-population of wt and KO THP-1 cells. Beta-actin: loading control. SAMHD1 was detected using a mouse polyclonal antibody **D**. Phosphorylated SAMHD1 was detected after electrophoresis in a phosgel using an antibody directed against pT592 (α-pSAMHD1) and a rabbit polyclonal antibody recognizing both the phosphorylated and the non-phosphorylated form (α-SAMHD1). Before electrophoresis, parallel samples were treated with (+) or without (-) lambda phosphatase (lambda PP). **E**. Comparison of dNTP pool sizes in wt and KO THP-1 cells in the indicated phases of the cell cycle. In the individual phases the level of each dNTP was calculated as detailed in Experimental procedures. The fold increase of each dNTP in KO cells relative to the wt is reported for each phase of the cycle. Bars: Mean ± standard deviation for n = 6 values. **F.** The composition of the dNTP pools in each phase of the cycle for wt and KO cells, evaluated from the ratios between pmoles of dATP, dTTP or dCTP and pmoles of dGTP under each condition.

It is still controversial if phosphorylation at T592 impairs SAMHD1 dNTPase activity, since *in vitro* data obtained with purified SAMHD1 variants do not match the effects of the same mutants on the dNTP pools of transfected cells [15,20–22]. Considering that overexpression of an ectopic protein *per se* might alter the physiological conditions, we chose to investigate the phosphorylation of the endogenous protein. We used THP-1 cells, a cell line spontaneously expressing high levels of SAMHD1 for which a SAMHD1 KO derivative was already available[23]. First, we assessed that the absence of SAMHD1 in KO cells did not affect cellular growth or cell cycle distribution (Figure 1(a)). Then, to detect the influence of SAMHD1 activity on dNTP levels during cell cycle progression, we compared the sizes of the four dNTP pools in the KO and parental THP-1 cells. To avoid possible alterations of the dNTP pools linked to chemical synchronization, we separated unperturbed cycling populations of the two lines by centrifugal elutriation and obtained fractions highly enriched in G1, S, and G2/M-phase (Figure 1(b)), from which we prepared samples for immunoblot and dNTP measurements. SAMHD1 was present in each phase of the cell cycle only in the parental THP-1 cells. The treatment of whole cell extracts with or without lambda-phosphatase, followed by phosgel electrophoresis, showed that SAMHD1 was phosphorylated at T592 in S and G2/M and dephosphorylated in G1 (Figure 1(c,d)). In both cell lines the R2 subunit of RNR was absent in G1 and equally expressed in the other phases, whereas the stable small subunit p53R2 and the long-lived large subunit R1 remained constant, indicating that DNA precursor synthesis by RNR was similar (Figure 1(c)). The presence of cyclin A2 and cyclin B in the elutriated fractions correlated with the expression patterns expected from the flow cytometry data. The four dNTPs were measured in all elutriated fractions and the size of each dNTP pool was calculated individually in G1, S and G2 as detailed in Experimental procedures (Figure 1(e)). The lack of SAMHD1 in the KO THP-1 cells was accompanied by enlarged dNTP pools in all phases, but the pools still fluctuated during cell cycle progression, indicating that the induction of RNR activity was not prevented by the unusually high concentrations of DNA precursors in G1 (Figure 1(e)). The fold increases of dNTP pools in THP-1 KO over control cells differed between nucleotides, with dTTP increasing relatively less than dGTP and dATP (Figure 1(e)). A comparison of relative compositions of the pools in each phase of the cycle in the two cell lines (Figure 1(f)) demonstrates that the larger pools of the SAMHD1 KO cells were imbalanced relative to the controls.

In proliferating cells the sizes of dNTP pools reflect the relative levels of synthesis, degradation and consumption for DNA synthesis. The two THP-1 lines grew at the same rate and with the same percentage of S-phase cells that expressed equal levels of RNR subunits. These features indicated that precursors synthesis by RNR and consumption for DNA replication were identical in the two lines during S-phase. If phosphorylation of T592 had inhibited SAMHD1 activity in wild type cells, their dNTP pools would have been similar to those in KO cells. Our data show that the pool differences between parental and SAMHD1 KO cells persisted during S-phase and peaked in G2/M, when DNA replication was over and RNR was still active due to the presence of R2 (Figure 1(e)). These observations strongly indicate that during the entire cell cycle SAMHD1 retained its dNTPase activity independent of phosphorylation at T592.

We wondered if other enzymes of dNTP catabolism were upregulated in the absence of SAMHD1, contributing to the containment of the dNTP pools. The activity of the major cytosolic 5ʹ-deoxynucleotidase (cdN) [24] was unchanged in whole cell extracts of KO and wild type THP-1 cells (measured specific activity 14.6 mU/mg in wild type and 15.4 mU/mg in KO cells), which suggests that SAMHD1 deficiency does not lead to induction of cdN expression. 5ʹ-nucleotidases are enzymes with relatively high k_m_s and their activities are regulated by the concentration of the substrates [24]. This mechanism is clearly not sufficient to maintain normal dNTP pool sizes in cells depleted of SAMHD1.

Our results show that the loss of endogenous SAMHD1 activity causes quantitative and qualitative alterations of DNA precursor pools during the entire cell cycle, indicating that the phosphorylation at T592 does not inactivate SAMHD1. Interestingly, with the exception of dCTP, the KO/control ratios of dNTP concentrations tended to be lower in S phase than in G1 and G2, which may reflect a partial attenuation of SAMHD1 activity during S phase.

### The phosphorylation of SAMHD1 T592 starts at the G1/S transition by CDK2 complexed with cyclin E or cyclin A2

**Figure 2.**
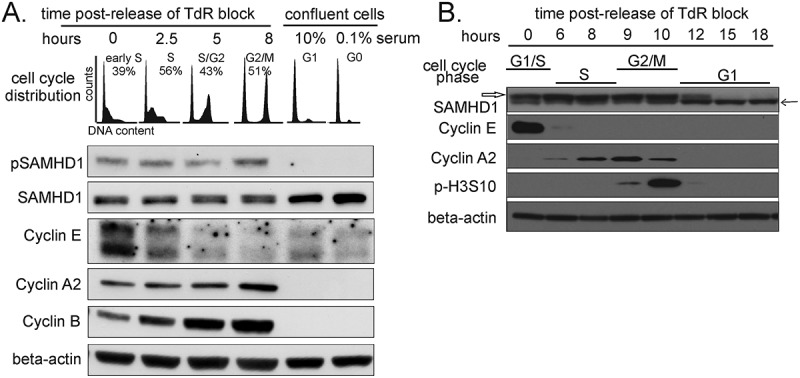
Phosphorylation status of SAMHD1 during cell cycle progression in normal human fibroblasts (A) and transformed cells (B). **A**. Cycling lung fibroblasts were synchronized by double thymidine block and released. Confluent fibroblasts were maintained for 48 h in medium with 10 or 0.1% serum. The top panel shows the cell cycle profiles at the indicated times after release and at confluence. The lower panels show the expression levels of SAMHD1 detected by an antibody directed against phosphoT592 (pSAMHD1) and a mouse monoclonal antibody recognizing both the phosphorylated and non- phosphorylated forms of SAMHD1 (SAMHD1). Cyclin E, A2 and B were used as cell cycle markers and beta-actin as a loading control. **B**. Whole cell extracts of HeLa cells synchronized by double thymidine block were analyzed for expression of SAMHD1 with the same mouse monoclonal recognizing both forms of SAMHD1. The phosphorylated form appears as a slower migrating band (empty arrow) compared to the non-phosphorylated form (black arrow). The cell cycle markers cyclin E, cyclin A and histone H3-S10-phosphorylation are those analyzed in the same experiment and data for these markers was published in Figure 2B of Kara et al [25].

Since we had found a sharp difference between G1 and S-phase in relation to T592 phosphorylation, we wished to define the timing of the appearance of pT592 in not transformed cells. We used a double thymidine block to obtain satisfactory synchronization of normal human lung fibroblasts at the G1/S border. After release of the block, the progression into S-phase was assessed by flow cytometry and by immunoblotting with several cell cycle markers (Figure 2(a)). As expected, cyclin E was present at the G1/S border and decreased as cells advanced into S phase, cyclin A2 accumulated during S and into G2, while cyclin B increased in G2/mitosis. SAMHD1 was present in all the phases and actually increased in confluent G1- and serum-starved G0 cells (Figure 2(a)), consistent with previous results[10]. In G1 and G0 T592 was not phosphorylated. The appearance of T592 phosphorylation correlated with the presence of cyclin E and cyclin A2 and it was maintained until mitosis. We confirmed this data in protein extracts of HeLa cells synchronized by double thymidine block, previously used to monitor the cell cycle-related assembly of origin recognition complexes [25], (Figure 2(b)).

**Figure 3.**
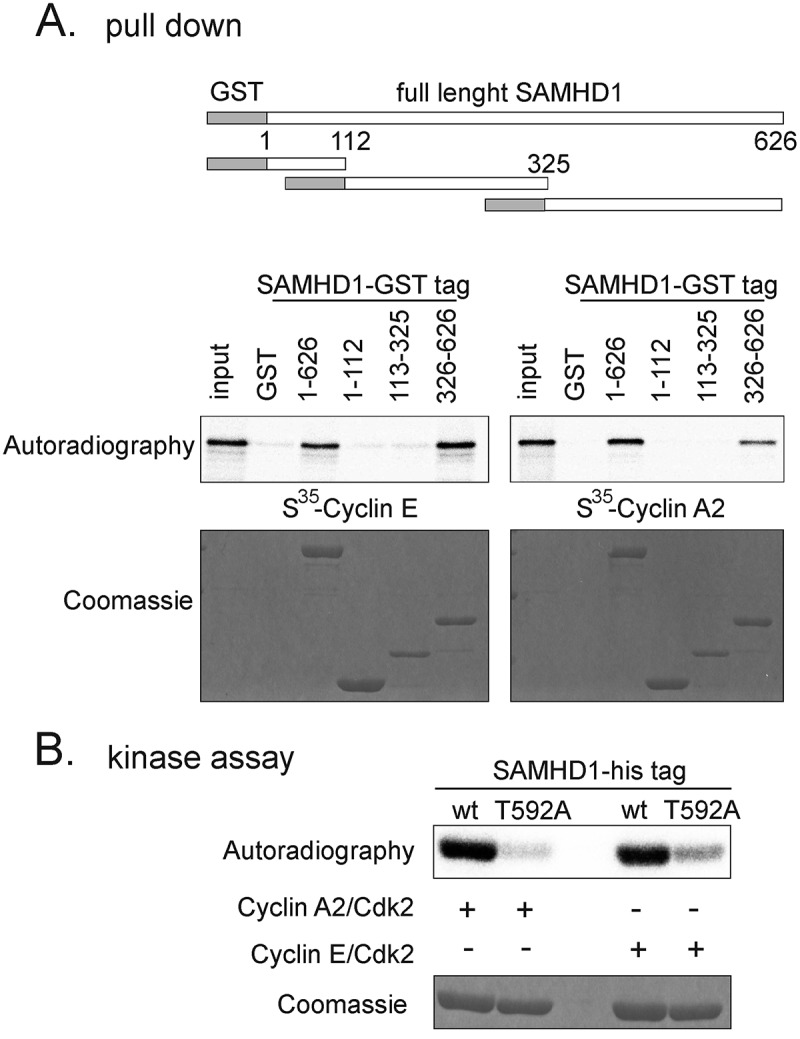
Cyclin E/CDK2 phosphorylates SAMHD1 *in vitro*. **A**. *In vitro* interaction between SAMHD1 and cyclin/CDK complexes. Top panel: schematic representation of GST-SAMHD1 constructs (GST in grey, SAMHD1 in white) used for *in vitro* pull down assays with cyclin E or cyclin A2. The full-length GST-SAMHD1 or the indicated fragments were bound to Glutathione agarose resin and then incubated with *in vitro* translated [S^35^]-labeled cyclin E or cyclin A2. Beads were isolated and bound proteins were separated by gel electrophoresis and visualized by autoradiography or Coomassie staining. **B**. *In vitro* kinase assay. Purified recombinant his- tagged wild type SAMHD1 (wt) or the non-phosphorylatable mutant T592A were incubated with recombinant cyclin A2/CDK2 or cyclin E/CDK2 in the presence of 1μCi [γ-^32^P]-ATP. Reactions were separated by SDS-PAGE and visualized by autoradiography or Coomassie staining.

We hypothesized that cyclin E complexed with CDK2 might be directly involved in SAMHD1 phosphorylation just before the onset of DNA replication. In order to further confirm our data we tested whether cyclin E, similar to cyclin A2 [14,16], interacts directly with SAMHD1. We prepared full-length or deletion derivatives of human SAMHD1 fused at the N-terminus with GST (upper panel of Figure 3(a)) and tested their ability to pull down *in vitro* translated S^35^-labeled cyclins E and A2. In agreement with previous results, full length SAMHD1 interacted with cyclin A2 and the C terminus was necessary for the interaction. Like cyclin A2, we found that cyclin E also bound to the C-terminus of SAMHD1 (lower panel of Figure 3(a)). In addition, when incubated with purified CDK2 complexed with either cyclin A2 or cyclin E, wild type SAMHD1, but not the T592A mutant, was efficiently phosphorylated by both kinases (Figure 3(b)). Together, the cell cycle synchronization experiments and the *in vitro* kinase assays suggest that the phosphorylation of SAMHD1 T592 is initiated before S-phase by the cyclin E-CDK2 complex and is maintained by cyclin A2-CDK2.

### SAMHD1 is a stable protein in cycling cells

**Figure 4.**
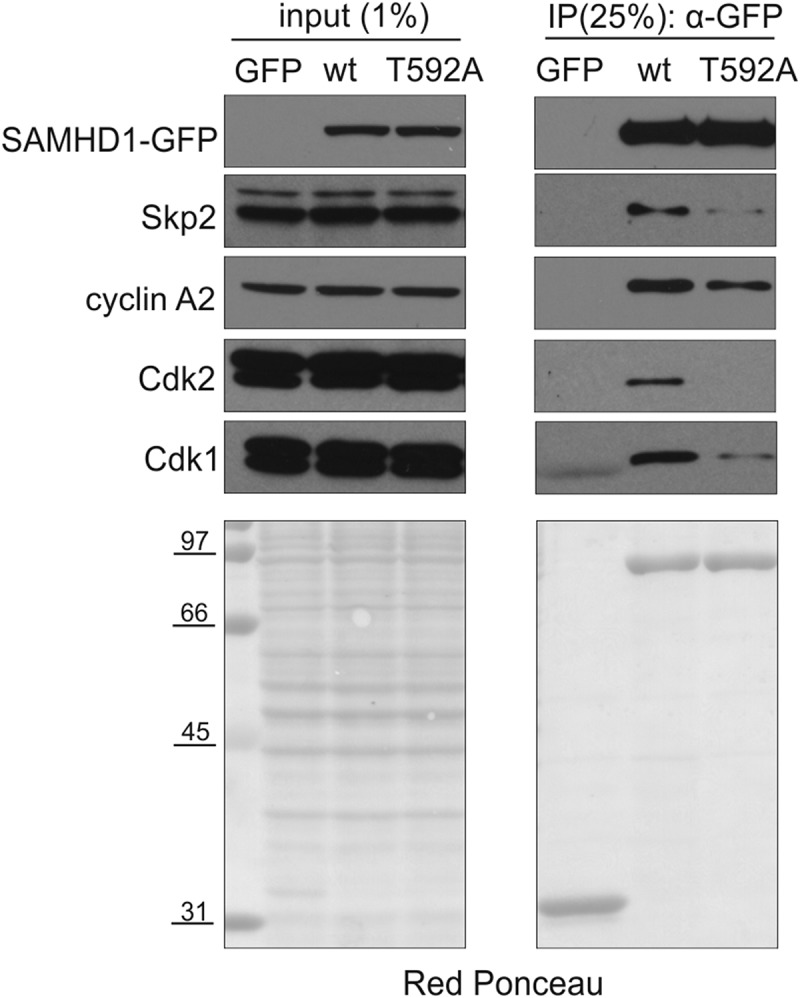
T592 phosphorylation favors SAMHD1 interaction with Skp2. U2OS cells were transiently transfected with a plasmid coding for GFP-tagged wild type SAMHD1 (wt) or non-phosphorylatable T592A SAMHD1 or the empty vector (GFP). After 48h cells were lysed and proteins immunoprecipitated (IP) from detergent extracts via the GFP tag. SAMHD1 and the indicated proteins were detected in immune-complexes and extracts by immunoblotting. Red Ponceau is used as loading control for protein extracts and shows the immune-complexes after IP.

Earlier reports identified SAMHD1 as an interacting partner of Skp2 in proliferating cells [16]. Skp2 is a component of the SCF ubiquitin ligase that recognizes phosphorylated proteins and regulates the proteolytic events driving cells through the G1/S transition. Considering the onset of T592 phosphorylation at the G1/S border, we asked whether phosphorylated SAMHD1 might be a substrate of the SCF ligase via Skp2 and be targeted to proteasomes. For this purpose we performed experiments in U2OS cells which can be efficiently transfected and have been previously used to study cell cycle related events. In co-immunoprecipitation experiments with extracts from U2OS cells transiently expressing GFP-tagged wild type or T592A SAMHD1, wild type SAMHD1 co-immunoprecipitated with Skp2 and other cell-cycle related markers such as cyclin A2, CDK2 and CDK1 (Figure 4). However, the interaction of the non-phosphorylatable T592A mutant with Skp2 and CDK2/1 was weaker, whereas the phosphorylation status of SAMHD1 only marginally affected the interaction with cyclin A2. Interestingly, CDK2 was not bound to the non-phosphorylatable SAMHD1, suggesting that active phosphorylation of SAMHD1 is required to establish complexes containing cyclin A2, CDK2 and Skp2 (Figure 4).

To determine whether the proteasome is involved in the degradation of endogenous SAMHD1 as cells enter S-phase, we treated human fibroblasts synchronized at the G1/S boundary with the proteasome inhibitor MG132 (Suppl Figure 1). We found that during S-phase a 2 h treatment with MG132 stabilized p53, leading to an increase of its transcriptional target p21, a potent inhibitor of CDK2, and to S-phase arrest. Accordingly, we found inhibition of SAMHD1 phosphorylation in the S-phase arrested cells (Suppl Figure 1 A-C).

To circumvent the cell cycle arrest due to MG132 treatment, we treated the synchronized fibroblasts with MLN4924. MLN4924 blocks the activation of cullin-RING ubiquitin ligases, including SCF^skp2^ and CRL4^Cdt2^ which both operate during S phase and could be involved in the degradation of phosphorylated SAMHD1. We found that MLN4924 treatment did not affect cell cycle progression or cause accumulation of SAMHD1 (Suppl Figure 1D), which suggested that in cycling cells SAMHD1 concentration is unlikely to be regulated by ubiquitin-dependent degradation.

**Figure 5.**
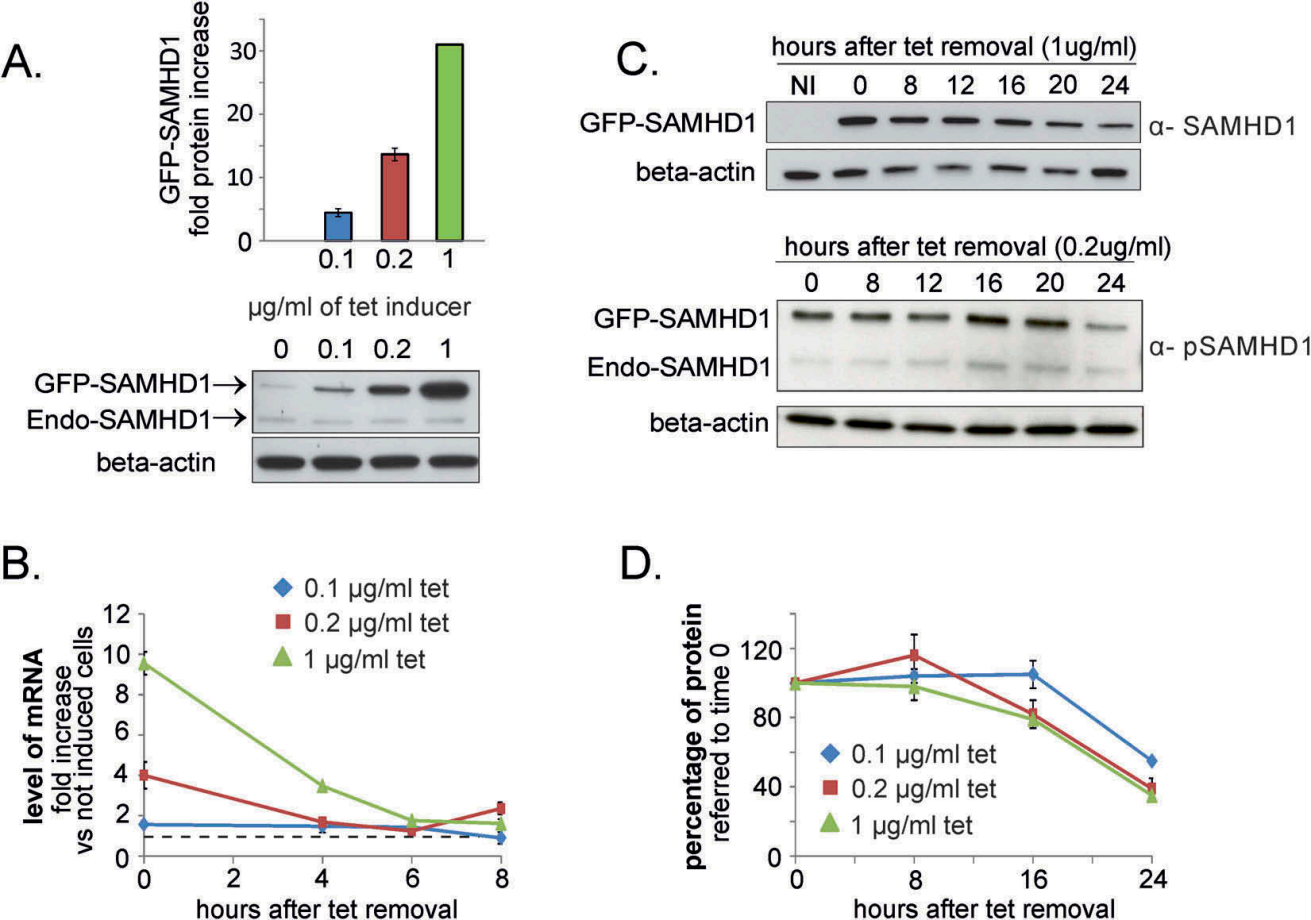
Turn-over of SAMDH1 in proliferating U2OS cells. **A**. The expression of GFP-tagged SAMHD1 was induced in U2OS cells stably transfected with a tetracyclin-inducible vector by treatment with different doses of tetracycline (tet) for 24 h and analyzed by immunoblotting. The lower panel reports a representative immunoblot of endogenous SAMHD1 (Endo-SAMHD1) and ectopically expressed GFP-SAMHD1. **B**. After tet removal the decline of the induced mRNA was followed by RT-PCR. The level of mRNA is reported as fold increase relative to that of not induced transfected cells (dashed line). **C**. The amount of GFP-SAMHD1 was determined by immunoblotting using antibodies against SAMHD1 and GFP. Representative immunoblots of total GFP-SAMHD1 (α-SAMHD1) and phosphorylated GFP-SAMHD1 (α-pSAMHD1) in samples induced for 24 h with 1 µg/ml tet and chased in the absence of tet for the indicated times. Beta actin: loading control. NI = not induced. **D**. Densitometric analysis was performed for GFP-SAMHD1, normalized for beta-actin and expressed as percentage relative to the protein level at 0 time. Bars in A.B. and D: Mean ± standard error, n = 3.

In order to further investigate whether SAMHD1 is subject to rapid proteolytic control, we determined the half-life of the protein. A U2OS cell line was constructed to express a tet-inducible SAMHD1 tagged with GFP at its N-terminus. These inducible cells were incubated with 0.1 to 1 µg/ml tetracycline for 24 h, resulting in a dose-dependent accumulation of the fusion protein (Figure 5(a)). Cell cycle progression was unaffected, despite the high level of ectopic SAMHD1 compared to the endogenous protein. The dGTP pool decreased (not shown), indicating that SAMHD1-GFP was catalytically active. After removing the inducer, we followed the disappearance of SAMHD1 mRNA and protein (Figure 5(b–d)). Irrespective of the dose of tet used, the mRNA declined rapidly and within 6–8 h reached a stable level close to that of the non-induced cells (Figure 5(b)), while the level of GFP-SAMHD1 halved only after 24 h (Figure 5(c,d)). Using both anti-SAMHD1 and anti-GFP antibodies we found no sign of degradation products linked to the overexpression. Considering the low level of mRNA after 8 h from tetracycline removal, we calculated for the protein a half-life of at least 16 h. We assessed the phosphorylation of GFP-SAMHD1 using the antibody specific for pT592 and found that the phosphorylated form decreased similarly to the non-phosphorylated one (Figure 5(c)). In line with our previous data with SAMHD1-silenced fibroblasts where the protein declined slowly in the absence of SAMHD1 mRNA [10], these results suggest that in proliferating cells SAMHD1 is a stable protein and phosphorylation does not fast SAMHD1 turn-over.

### SAMHD1 is dephosphorylated during mitotic exit in parallel with pThr-CDK substrates

As cells exit mitosis, CDK1 is inhibited and most of its substrate proteins are dephosphorylated by protein phosphatases [26]. Since phosphorylation of T592 depends on CDK2/1 activity, persists in mitosis and is absent in G1 (Figure2(a,b)), we asked whether SAMHD1 was dephosphorylated before the cell divides. To study the events taking place during mitosis, we turned to human retinal cells (hRPE-1 line) that can be efficiently synchronized in mitosis.

**Figure 6.**
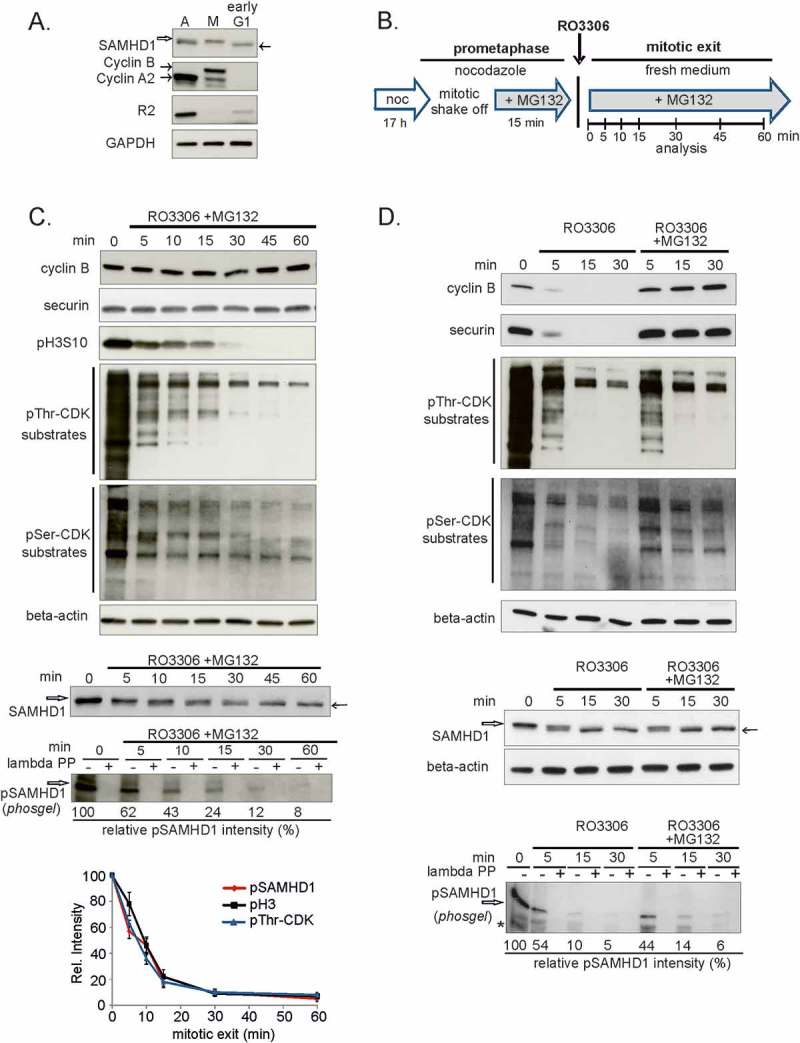
Kinetics of SAMHD1 dephosphorylation during mitotic exit in hRPE-1 cells. **A**. hRPE-1 cells were synchronized in prometaphase by 16 h nocodazole treatment (100 ng/ml), mitotic cells were detached by gentle shake-off and seeded in fresh medium. Samples were collected without nocodazole synchronization (A), immediately after shake-off (M) and after 2 h in fresh medium (early G1) and analyzed by immunoblotting. SAMHD1 was detected by a mouse monoclonal antibody recognizing both the phosphorylated (empty arrow) and the non-phosphorylated form (black arrow) as indicated by a band shift. Cyclin A2, cyclin B and R2, the S-phase induced small subunit of ribonucleotide reductase, are used as cell cycle markers. GAPDH: loading control. **B**. Schematic diagram of the protocol used to synchronize mitotic exit in hRPE-1 cells. Samples were collected in prometaphase and during mitotic exit at the indicated time points after RO3306 addition. The proteasome inhibitor MG132 was present 15 min before RO3306 addition and during mitotic exit. **C**. hRPE-1 cells were synchronized as in B and whole cell extracts were immunoblotted for cyclin B, securin, phospho-Histone H3(Ser10) (pH3S10), pThr-CDK substrates, pSer-CDK substrates and beta-actin (loading control). In the same experiment SAMHD1 phosphorylation was analyzed as in A. by electrophoresis in a phosgel using the antibody against phospho T592 (pSAMHD1) after pre-treatment in the presence (+) or absence (-) of lambda phosphatase (PP). Empty arrow: pSAMHD1, black arrow non-phosphorylated SAMHD1. Decay of protein phosphorylation was evaluated from the relative intensities of pSAMHD1, pThr-CDK substrates and pH3S10 normalized for beta-actin and the level of phosphorylation at 0 min, taken as 100%. Bars: Mean ± standard error for n = 4 values. **D**. hRPE-1 cells were synchronized as in B. After RO3306 addition one set of cultures was treated with proteasome inhibitor MG132 (RO3306 +MG132). Samples were collected at 0–5-15–30 min during mitotic exit and immunoblotted for cyclin B, securin, pThr-CDK substrates, pSer-CDK substrates and beta-actin (loading control). SAMHD1 was analyzed as described in C. *non-specific band

First we followed the presence of pT592 SAMHD1 in hRPE-1 cells synchronized in mitosis by nocodazole treatment and then released to allow mitotic exit and transition into G1-phase. The presence of the markers cyclin A and B and the absence of the R2 subunit of RNR demonstrated that the cells had completed G2 and were in mitosis, whereas the entrance into early G1 phase was demonstrated by the disappearance of these three markers and the doubling of cell number from 1.5 million to 3.3 million cells within 2 h after re-plating the isolated mitotic cells. SAMDH1 was phosphorylated in nocodazole-arrested hRPE-1 cells and was completely dephosphorylated soon after cytokinesis (Figure 6(a)).

Then we examined the timing of SAMHD1 dephosphorylation during synchronized mitotic exit obtained by a two-step synchronization protocol [27, 28]. Briefly, cultures were first enriched of mitotic cells by overnight treatment with nocodazole. Prometaphases were collected through gentle mitotic shake-off and re-plated. Then the CDK1 inhibitor RO3306 (Figure 6(b)) was added to induce a highly synchronized mitotic exit in the presence of MG132 to minimize the loss of phosphorylated proteins via degradation. We applied this protocol to hRPE-1 cells and collected samples for immunoblotting. Securin and cyclin B levels were monitored as indicators of APC/C^cdc20^ activity, phospho-histone H3 (Ser10) (pH3S10) was monitored as a marker of condensed mitotic chromosomes, and we followed the dephosphorylation of CDK substrates with anti phospho-threonine (pThr-CDK) and anti phospho-serine (pSer-CDK) antibodies. After RO3306 addition, securin and cyclin B remained stable due to the presence of the proteasome inhibitor, while the disappearance of pH3S10 marked the entrance into anaphase [29], (Figure 6(c)). Significant dephosphorylation of pThrCDK sites occurred within 15 min after RO3306 addition, whereas dephosphorylation of pSerCDK sites was slower, reflecting differential dephosphorylation patterns during mitotic exit [27]. Turning to SAMHD1, dephosphorylation was apparent within 15 min (Figure 6(c)) showing the same pattern of dephosphorylation of pThr-CDK substrates (lower panel in Figure 6(c)). This rapid dephosphorylation of SAMHD1 was confirmed using phosgel with an antibody specific for the phosphorylated form. Using the same protocol we evaluated SAMHD1 dephosphorylation during mitotic exit in the tumor cell line U2OS and confirmed that the pattern of dephosphorylation of SAMHD1 paralleled that of pThr-CDK substrates (Supp Figure 2).

Considering that some phosphorylated proteins may be degraded in proteasomes during mitotic exit, we repeated the experiment monitoring SAMHD1 in the presence or absence of MG132 (Figure 6(d)). The persistence of APC/C^cdc20^ substrates (securin and cyclin B) assessed the effectiveness of the MG132 treatment during mitotic exit. The kinetics of dephosphorylation of both pThrCDK and pSerCDK sites were not affected by the block of proteasome activity (Figure 6(d)). Immunoblotting for SAMHD1 with both the antibody detecting total SAMHD1 and the antibody specific for pT592 after phosgel electrophoresis showed that neither the timing of dephosphorylation nor the amount of pSAMHD1 were influenced by proteasome inhibition, suggesting that protein degradation did not play a role in regulating SAMHD1 at the end of mitosis.

Our data indicate that SAMHD1 is one of the target proteins that must be dephosphorylated in preparation for the G1 phase.

### The dephosphorylation of SAMHD1 depends on the activity of different phosphatases

During mitotic exit the rise of phosphatase activities combined with the decline of kinase activities re-establish the low level of protein phosphorylation characteristic of G1 cells. In yeasts Cdc14 is the primary phosphatase counteracting CDK1 activity. In human cells Cdc14 does not appear to play a central role, but protein phosphatases of the PP1 and PP2A superfamilies are the major enzymes that reverse CDK1 action [26]. We wondered whether Cdc14 or members of the PP1–2 superfamilies were involved in SAMHD1 dephosphorylation during mitotic exit. We compared the timing of pSAMHD1 dephosphorylation in a hRPE-1 line KO for the Cdc14A isoform [30] and in its parental line, but did not detect any difference between the two lines (data not shown). It is therefore unlikely that Cdc14A dephosphorylates SAMHD1 during mitotic exit, even if we cannot exclude some compensatory activity by isoform Cdc14B.

**Figure 7.**
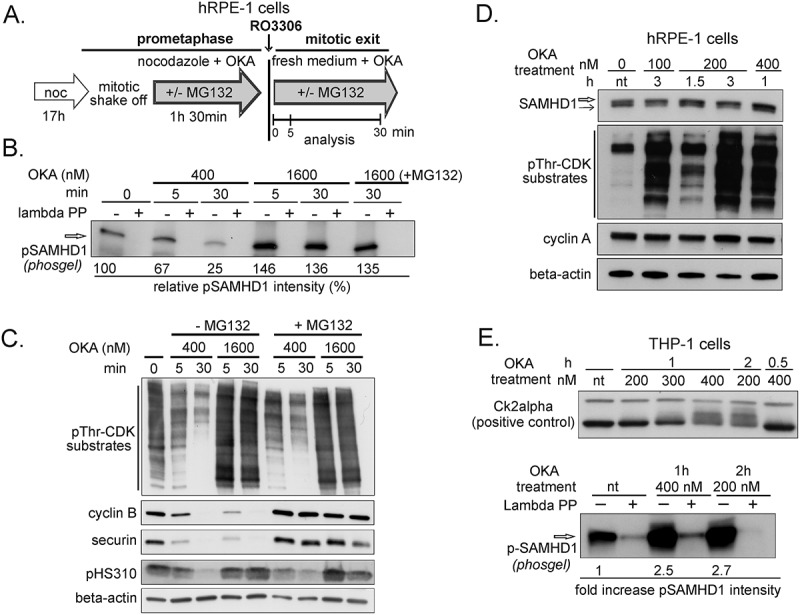
Okadaic acid treatment maintains SAMHD1 phosphorylation in normal and transformed cells. Okadaic acid was added to hRPE-1 cells during synchronous mitotic exit (A-C), and to asynchronous populations of hRPE-1 (D) or THP-1 cells (E). **A**. Schematic diagram of the experiment for hRPE-1 cells. Prometaphases of nocodazole synchronized cells were incubated with okadaic acid (OKA) before RO3306 addition and during mitotic exit in the presence (+MG132) or absence (-MG132) of proteasome inhibitor MG132. Samples were collected at the indicated times. **B**. hRPE-1 cells were synchronized and treated with 400 or 1600 nM OKA as indicated in A. Whole cell extracts were analyzed by immunoblotting using the antibody against phospho T592 (pSAMHD1) after pre-treatment with (+) or without (-) lambda phosphatase (PP) and electrophoresis in a phosgel. **C**. The mitotic markers (pThr-CDK substrates, cyclin B, securin, pH3S10) and loading control (beta-actin) were analyzed in parallel. **D**. asynchronous proliferating hRPE-1 cells and **E**. THP-1 cells were treated with OKA as indicated. Whole cell extracts were analyzed for pThr-CDK substrates or for CK2alpha kinase as positive controls for OKA treatment. SAMHD1 was detected either by an antibody recognizing both the phosphorylated (empty arrow) and the non-phosphorylated form (black arrow) or by anti pSAMHD1 antibody after electrophoresis in a phosgel after pretreatment with (+) or without (-) lambda phosphatase (PP).

Next we investigated if the major mitotic phosphatases, PP1 and PP2A, are involved in SAMHD1 dephosphorylation. The two classes of enzymes can be distinguished on the basis of their differential sensitivity to the inhibitor okadaic acid (OKA)[31]. We treated prometaphase hRPE-1 cells with OKA before and after RO3306 addition and collected samples for immunoblotting (Figure 7(a)). Compared to no OKA treatment, the disappearance of pT592 was only slightly delayed by the lower dose of OKA (400 nM) whereas with the higher dose (1600 nM) the pT592 signal persisted and actually increased, independent of the presence of MG132 (Figure 7(b)). During the treatments with OKA the pT592 signal changed in parallel with that of pThr-CDK substrates. Securin and cyclin B disappeared rapidly while the decline of pH3S10 and pThr-CDK substrates was OKA dose dependent (Figure 7(c)). Considering that in human cells OKA doses below 1 µM are reported to be ineffective against PP1 phosphatases [32,33], our data point to an involvement of phosphatase(s) of the PP1 superfamily in the dephosphorylation of SAMHD1 during mitosis.

We asked if the level of pT592 is modulated by phosphatase activity also during S-phase. For this purpose, asynchronous proliferating hRPE-1 cells, containing only a small fraction of mitoses, were treated with OKA for different times. We found that a low concentration of OKA (100–400 nM) increased pSAMHD1 over the non-phosphorylated form (Figure 7(d)). Similarly, in asynchronous THP-1 cultures (≥40% S-phase) analogous concentrations of OKA induced a marked accumulation of pSAMHD1 (Figure 7(e)) indicating that in cycling cell populations SAMHD1 is actively dephosphorylated by PP2 phosphatases.

Taken together our results indicate that the phosphorylation state of SAMHD1 at T592 is finely regulated and results from the interplay between kinase and phosphatase activities controlling events important in cell cycle progression.

## Discussion

In mammalian cells the concentrations of deoxynucleotides are adjusted to the requirements of nuclear DNA synthesis. RNR and SAMHD1 are the major regulators of the intracellular concentrations of dNTPs. In S-phase the induction of the R2 subunit of RNR and the phosphorylation of SAMHD1 at T592 by the S-phase CDK complexes suggested a concerted regulation between the two opposite enzymatic activities. In our previous work [10] we suggested that SAMHD1 exerts its activity mainly in G1-phase and in quiescent cells. Here we show that SAMHD1 regulates the DNA precursors during the entire cell cycle. In particular, we find that SAMHD1 is still active in S-phase when it is totally phosphorylated at T592.

Indirect evidence that SAMHD1 is functional even in cycling cells with high dNTP pools comes also from two recent reports that tested the sensitivity to some nucleoside analogs in cancer cells and in cells permissive to HIV-1 infection [34,35].

While it is understandable why SAMHD1 may be useful to prevent an accumulation of dNTPs outside S-phase, it is unclear why SAMHD1 should degrade DNA precursors when they are needed during genome replication. Considering that a surplus of dNTPs is detrimental to the fidelity of DNA replication in both bacteria and eukaryotes [36–38], we hypothesize that SAMHD1 activity in S-phase is essential to contain the huge expansion of DNA precursors due to the induction of the ribonucleotide reductase subunit R2 and to maintain balanced dNTP pools in cooperation with RNR activity. Catabolic SAMHD1 and anabolic RNR show striking similarities in their allosteric properties, with allosteric sites binding dNTPs and controlling the activity at the catalytic site. It is intriguing that the same nucleotide, dATP, is a feed-back allosteric inhibitor of RNR, switching off the production of cellular dNTPs, and an efficient allosteric activator of dNTP degradation by SAMHD1 [1,39]. Such coordinated allosteric feedback inhibition of both enzymes would maintain dNTP levels at a proper and balanced level during DNA replication.

SAMHD1 is phosphorylated at different sites, but only pT592 was found to be related to the state of cellular growth, being present only in proliferating cultures [15,21]. Previous work by Yan et al. showed that pT592 is present in S and G2/M in subpopulations of leukocytes isolated by cell sorting [18]. Here we followed the phosphorylation pattern of T592 during cell cycle progression in synchronized living cells and found that it is intimately linked to kinase and phosphatase activities controlling cell cycle progression.

We propose an involvement of Cyclin E/CDK2 in SAMHD1 phosphorylation which occurs just before DNA replication begins. Cyclin E is regulated by E2F and controls DNA replication at the G1/S transition, stimulating the assembly of replication complexes and licensing of replication origins, whereas cyclin A activates origin firing and DNA synthesis [40]. Substrate specificities of cyclins E and A overlap [41], which accounts for the participation of both cyclins to SAMHD1 phosphorylation.

During mitotic exit a timely dephosphorylation program coordinates and regulates the events in anaphase, telophase and cytokinesis leading to proper cell division. In our analysis we found that the absence of pT592 in G1-phase is due to phosphatase(s) acting in parallel on SAMHD1 and on the bulk of CDK target proteins during mitotic exit. It remains to be investigated why SAMHD1, an enzyme involved in the control of the cellular concentrations of DNA precursors is one of the phosphatase substrates during mitotic exit.

We tried to identify the phosphatase involved in the process using chemical inhibition, because individual knockdown of phosphatases causes cell cycle arrest at earlier stages of mitosis. We took advantage of the 10–100 fold differential selectivity of the cell-permeable inhibitor okadaic acid (OKA) for PP2A over PP1 with active concentrations below 1 µM reported as specific for PP2A in human cell lines [32,33]. The requirement for ≥1 µM OKA to fully inhibit SAMHD1 dephosphorylation during mitotic exit suggests the involvement of the PP1 family of phosphatases. In addition, the absence of a recognition sequence for phosphatase PP2/B55 substrates[42] (a bipartite positive-charged polybasic motif surrounding the central CDK consensus site) in human SAMHD1 protein provides a further evidence for this hypothesis.

Interestingly, we noticed the that okadaic acid was effective at doses below 1 µM in asynchronous populations with ≤10% mitotic cells. This finding indicates an involvement of PP2A phosphatases outside mitosis and suggests that different phosphatases are implicated in SAMHD1 dephosphorylation in different cell cycle phases. These experiments, however, did not allow the identification of specific members of the two super-families acting on SAMHD1.

Earlier, we suggested that T592 phosphorylation may be a signal for SAMHD1 degradation during S-phase [10,43]. Here we showed that SAMHD1 co-immunoprecipitates with Skp2 in a phosphorylation dependent manner but we could not detect an increase of SAMHD1 protein after inhibition of the proteasome or of protein neddylation. Moreover, we could not determine accurately the abundance of SAMHD1 in the individual phases of the cycle in synchronized or elutriated cells since the results obtained by immunoblotting were strongly influenced by the type of antibody (mouse or rabbit) and the specific batch used for the analysis. In protein extracts treated ± lambda phosphatase we found that the intensities of the phosphorylated and non-phosphorylated bands in the untreated samples did not add up in the single non-phosphorylated band recovered after the treatment. Therefore, we investigated the turn-over of SAMHD1, that might reveal a rapid proteolytic control. Ectopic expression of GFP-SAMHD1 was induced in actively diving U2OS cells (≈ 40% in S-phase) and the protein half-life was measured during a chase in the absence of the inducer. In contrast to a recent publication [44] but in agreement with our previously published data [10], we found that SAMHD1 behaves as a stable protein in proliferating cells, suggesting that its phosphorylation is unlikely to trigger rapid degradation.

The question of why SAMHD1 is phosphorylated remains open. Remarkably, the timing of SAMHD1 phosphorylation is the same in normal and tumor cells, suggesting a general regulatory mechanism, independent of cell transformation. Phosphorylation may modify the interactions of the protein with some as yet unidentified factor, modulating SAMHD1 functionality or sub-nuclear localization without turning off enzyme activity. On the other hand, we observed that the SAMHD1 mutant that cannot be phosphorylated still binds to cyclin A but it fails to co-immunoprecipitate the ubiquitin ligase subunit SKP2. Based on this observation, we suggest that phosphorylated SAMHD1 could form an active ubiquitin-ligase complex with SKP2 or Cyclin F, a related F-box protein, to control the level of a protein associated with SAMHD1. Alternatively, SAMHD1 phosphorylation may have no specific function, but be a side-effect of CDK activation at the onset of S-phase. We are examining these possibilities.

### Experimental procedures

#### Cell lines and cell growth

Human lung fibroblasts (CCDLu34) from the American Type Culture Collection were maintained in DMEM with 4.5 g glucose/L (Gibco Cat. 31,966–021) + 10% (vol/vol) FCS + nonessential amino acids + 20 mM Hepes buffer pH 7.4. Human retinal epithelial cells immortalized with hTERT (hRPE-1) knock-out for Cdc14A and parental control cells were a gift of Dr Elmar Schiebel and were grown in DMEM/F-12 (1:1) (Gibco Cat. 11,320–074) + 10% (vol/vol) FCS. Human monocytic cells (THP-1) knock-out for SAMHD1 and matched controls were donated by T. Gramberg and cultured in RPMI (Gibco Cat. 61,870–010) with + 10% (vol/vol) FCS. Human osteosarcoma cells (U2OS) were grown in DMEM with 4.5 g glucose/L + 10% (vol/vol) FCS. All cell lines were tested negative for the mycoplasma contamination.

U2OS were either transiently transfected with the constitutive expression vector pEGFP-C1 coding for SAMD1 (see Immunoprecitation) or stably transfected with a vector for Tet-inducible overexpression of wild type GFP- SAMHD1 to study the turn-over of SAMHD1 protein. Briefly, human wild type GFP- SAMHD1 was cloned into plasmid pcDNA 5/FRT/TO (Life Tecnologies) under the control of a tetracycline-regulated CMV-based promoter. The plasmid was integrated into FRT-U2OS-TRex osteosarcoma cells via FRT-mediated recombination, and the integrated transgene was selected with hygromycin. Expression of GFP-SAMHD1 was assessed following treatment of cells with various concentrations of tetracycline.

#### Reagents and antibodies

Thymidine (Sigma Cat. T9250), Nocodazole (Sigma Cat. M1404), MG132 (Selleckchem Cat. S2619), Okadaic acid (AdipoGen Cat. AG-CN2-0056), RO3306 (Sigma Cat. SML0569), Hygromycin B (Invitrogen Cat. 10,687–010), Tetracycline (Sigma Cat.T7660). The primary antibodies directed against the following proteins were: anti-SAMHD1 (mouse monoclonal Abcam, Cat. ab128107; mouse polyclonal Abcam, Cat. ab67820; rabbit polyclonal Proteintech, Cat. 12,586–1-AP), phospho-Thr592 SAMHD1 (rabbit polyclonal ProSci, Cat. 8005), anti-Cyclin A2 (mouse monoclonal Cell Signaling, Cat. 4656), anti-Cyclin B1 (mouse monoclonal Santa Cruz, Cat. Sc-245), anti-Cyclin E (rabbit monoclonal Abcam, Cat. Ab33911), anti-beta actin (mouse monoclonal Sigma Cat. A5316), anti-Skp2 (rabbit polyclonal Santa Cruz, Cat. Sc-7164), anti-Securin (mouse monoclonal Abcam, Cat. Ab3305), anti-GFP (rat monoclonal Cromotek, Cat. ABN670671), anti-phospho-Histone H3 Ser10 (rabbit polyclonal Cell Signaling, Cat. 9391), anti-phospho-Threonine-Proline (mouse monoclonal Cell Signaling, Cat. 9391), anti-phospho-CDK substrate motif (k/H)pSP (rabbit monoclonal Cell Signaling, Cat. 9477), anti WAF1/p21 (mouse monoclonal Calbiochem, Cat. OP64), anti-R2 (goat polyclonal Santa Cruz, Cat. sc-10,844), anti-R1 (mouse monoclonal Millipore, Cat. JC1650209), anti-p53R2 (goat polyclonal Santa Cruz, Cat. sc-10,840), anti-CK2α kindly donated by prof. A. Donella (Department of Molecular Medicine, University of Padova).

#### Cell cycle synchronization

For synchronization at the G1/S border, CCDLu34 were cultured in the presence of 2 mM thymidine for 16 h, washed with warm medium and maintained in fresh medium without TdR for 8 h. After additional 16 h in thymidine, cells were washed and released in fresh medium. To trap RPE-1 and U2OS cells in metaphase, 100 ng/ml nocodazole was added to fresh medium. After 17 h of incubation floating cells were removed by shake-off, centrifuged and incubated in fresh medium with 10 µM MG132 for 15 min. Then mitotic cells were forced to undergo mitotic exit by addition of 10 µM RO3306.

#### Immunoblotting

Pellets of 1-2 million cells were washed with PBS and lysed in radio-immunoprecipitation assay (RIPA) buffer (10 mM Tris-HCl, pH 7.4, 100 mM NaCl, 1% sodium deoxycholate, 0.1% SDS, 1% Nonidet P-40) containing a mixture of protease and phosphatase inhibitors for mammalian cells (Roche Applied Science). The extracts were incubated at 4°C for 30 min and then centrifuged at 19,000 × *g* for 10 min. The protein concentration of cleared supernatants was determined by the BCA protein assay (Pierce). For phos-histone H3 (ser10) detection the proteins were extracted in Laemmli buffer and boiled for 10 min. Equal amounts of protein were loaded in 7.5% or AnyKD precast gels (Bio-Rad), electrophoresed and blotted on Hybond-C extra (GE Healthcare Life Sciences). The membranes were saturated for 1h at room temperature with 2% non-fat milk (Euroclone); membranes for phosphorylated proteins were saturated with 2% ECL Blocking Agent (GE Healthcare Life Sciences) with 1% polyvinylpyrrolidone (PVP). Primary antibodies were incubated overnight at 4°C and horseradish peroxidase-conjugated secondary antibodies for 1 h at room temperature. Development was performed using a chemiluminescence ECL kit (LiteAblotTurbo, Euroclone) and the signals were detected on autoradiographic films (GE Healthcare Life Sciences). Densitometry was performed by ImageJ software.

#### Lambda phosphatase treatment and SDS-PAGE on phos-tag gels

To remove phosphate groups from serine, threonine and tyrosine residues 40 µg of the same cell extracts used for immunoblotting were treated with 0 or 400 U of Lambda Protein Phosphatase (λPP) kit (New England Biolabs Inc.) for 90 min at 30°C as specified in the manufacturer’s protocol. After adding Laemmli buffer, samples were boiled for 5 min and loaded on a 7.5% precast polyacrylamide SuperSep^TM^ Phos-tag^TM^ gel (Wako Chemicals USA, Cat. 195–17,371). Just after the electrophoresis the gel was soaked in a general transfer buffer containing 5 mM EDTA (10 min x 3) to eliminate the Zn^++^ ions. Next, the gel was soaked in a general transfer buffer without EDTA for 20 min and then electroblotted on a PVDF membrane. The blot was saturated for 1 h at room temperature with 2% ECL Blocking Agent (GE Healthcare Life Sciences) and 1% polyvinylpyrrolidone (PVP). Then the membrane was incubated and developed as described above.

#### Elutriation procedure

A suspension of about 90 million cells, chilled on ice and suspended in 8 ml of ice-cold RPMI containing 5% fetal calf serum, was injected in the elutriation chamber of a Beckman Avanti J-25 high performance centrifuge equipped with a JE-6B rotor, a standard chamber and a Cole-Parmer system model 7553–75 pump. The centrifuge was run continuously at 2,500 rpm. Separate 100 ml fractions were collected by pumping ice-cold RPMI with 5% fetal calf serum through the chamber at increasing flow rates (from 26 to 50 ml/min). The first and the last fraction were discarded. Each elutriated fraction was immediately centrifuged at 4 °C for 10 min at 300 × *g* and washed with cold PBS. Cells were counted in a Coulter Z1 counter, portions of about 10^6^ cells each were taken for flow cytometric analysis, nucleotide pool determination and immunoblotting. Number of experiments for each cell line = 3.

#### Flow cytometric analysis of cell cycle

The distribution of cells in the different phases of the cell cycle was determined by flow cytometry in a FACSCanto™ II flow cytometer (Becton Dickinson), equipped with a 488-nm argon ion laser. The cells were harvested, washed twice in PBS and fixed in 70% ethanol overnight at 4°C. Cells were then centrifuged, washed and incubated with PBS containing 50 µg/ml propidium iodide and 100 μg/ml RNase A (Sigma-Aldrich) for 1 h at 37°C. DNA fluorescence was measured in 25,000 cells.

#### Quantification of dntp pool sizes in G1, s and G2 by DNA polymerase assay

Extraction of the soluble nucleotide pool and determination of dNTP pool sizes were performed as previously described[45]. The sizes of the 4 pools in G1, S and G2 were calculated in the elutriated fractions as described by Bianchi et al [46] .. Briefly, the pool sizes in G1 (*a*) were measured in the fraction containing ≥98% of G1 cells; pool sizes in S (*b*) were determined in fractions enriched in S and devoid of G2 cells (typically 2–3 fractions per experiment) using this equation (*a* x % cells in G1) + (*b* x % cells in S) = 100 x dNTP pool size_det_. Once *a* and *b* were known the G2 pool sizes (*c*) were calculated in G2 enriched fractions devoid of G1 from the equation (*b* x % cells in S) + (*c* x % cells in G2) = 100 x dNTP pool size_det_. Number of experiments for each cell line = 3.

#### Recombinant protein expression and purification

Wild type and T592 mutant his-tagged SAMHD1 was produced and purified as described previously45. Full-length SAMHD1 and its deleted derivatives were fused to Glutathione S transferase (GST) by cloning into the bacterial expression vector PGEX6P1 (GE Healthcare Life Sciences). The GST fusion proteins were expressed and purified using Glutathione sepharose beads according to the procedure described previously[47].

#### Kinase assay

Cyclin E/CDK2 and Cyclin A/CDK2 were expressed in recombinant baculovirus infected Hi5 insect cells and purified as in Hossain and Stillman [48]. The phosphorylation of 1 µg of wild type and T592 mutant his-tagged SAMHD1 was carried out in the presence of 10 nM of purified CyclinA/CDK2 or CyclinE/CDK2 in a 15 µl reaction containing 0.1 mg/ml BSA, 0.2 mM cold ATP and 1 µCi [γ-P^32^]-labeled ATP in reaction buffer (50 mM Tris-HCl pH 7.5, 10 mM MgCl_2_, 1 mM DTT) for 30 min at 37°C. The reaction was quenched with Laemmli buffer and boiled for 5 min. Proteins were separated by SDS-PAGE, the gel was stained with Coomassie Brilliant Blue and autoradiographed.

#### Pull down *assay*

The GST and GST-SAMHD1 peptides were bound to Gluthatione agarose resin (Pierce) and the beads were washed three times with binding buffer (25 mM Tris pH7.5, 100 mM KCl, 0.1% NP-40, 0.1 mM EDTA, 5 mM MgCl, 1 mM DTT, 10% Glycerol with addition of protease inhibitors). [S^35^]-labeled cyclin A2 and cyclin E were produced using Quick TNT-coupled reticulocyte lysate system (Promega) according to the manufacturer’s instruction in the presence of [S^35^]-methionine (PerkinElmer). For each reaction 8 µl of [S^35^]-labeled cyclin proteins and the beads containing 3 µg of GST-tagged protein were incubated in 400 µl of binding buffer overnight at 4°C. The resin with bound proteins was washed four times with binding buffer and the proteins were eluted in Laemmli buffer and analyzed by SDS-PAGE followed by PhosphorImaging analysis.

#### Immunoprecipitation

U2OS cells were transfected with 15 μg of plasmid pEGFP-C1 (empty vector or coding for wild type or T592A mutant SAMHD1) using Lipofectamine2000 (Invitrogen) according to the manufacturer’s instructions. At 48 h post-transfection cells were collected for immunoprecipitation which was performed using standard protocol with the following modifications. For each transfection 10 million cells were lysed in 500 μl lysis buffer (0.4% NP-40, 100 mM NaCl, 20 mM Tris- HCl pH 7.5, 1 mM DTT, 2 mM CaCl_2_, Benzonase, 10 µM MG132, protease and phosphatase inhibitors) for 30 min in ice. After centrifugation at 14,000 g for 15 min at 4°C the supernatant was diluted with 500 μl of dilution buffer (20 mM Tris-HCl pH7.5, 10% glycerol, 1 mM DTT, 5 mM MgCl_2_, 0.1 mM EDTA) and centrifuged again. The supernatant was immunoprecipitated with 10 μl of cross-linked 4% agarose beads GFP-trap_A (Chromotek) for 2 h at 4°C under rotation. The precipitants were washed three times with washing buffer (20 mM Tris-HCl pH7.5, 0.1% NP-40, 50 mM NaCl, 10% glycerol, 1 mM DTT, 5 mM MgCl_2_, 0.1 mM EDTA). The immune-complexes were boiled with Laemmli buffer for 5 min and analyzed by SDS-PAGE followed by immunoblotting.

#### Quantitative real-time PCR

Relative level of SAMHD1 mRNA was determined by RT-PCR using Applied Biosystem 7500 Real Time PCR System (Applied Biosystems). Total RNA was extracted with TRIzol reagent (Invitrogen), cDNAs prepared by reverse transcription and real-time PCR performed in 96-wells optical plates as described by Franzolin et al [10]. Each cDNA preparation was analyzed at least six times.

#### Deoxynucletidase enzymatic assay

Total 5ʹ-deoxynucleotidase (cytosolic + mitochondrial deoxynucleotidase) activity was determined in crude whole cell extracts with 5 mM [H^3^]-labeled dUMP as substrate, as described by Rampazzo et al [49]. The enzymatic specific activity is reported as mU x mg prot^−1^ (1 mU = 1nmol product/min^−1^).

## References

[1] Ji X, Tang C, Zhao Q, et al Structural basis of cellular dNTP regulation by SAMHD1. Proceedings of the National Academy of Sciences [Internet]; 2014; 111:E4305–14. Available from: http://www.pnas.org/cgi/doi/10.1073/pnas.1412289111 10.1073/pnas.1412289111 PMC4205617 25267621

[2] Tüngler V, Staroske W, Kind B, et al Single-stranded nucleic acids promote SAMHD1 complex formation. J Mol Med. 2013;91:759–770. 23371319 10.1007/s00109-013-0995-3

[3] Beloglazova N, Flick R, Tchigvintsev A, et al Nuclease activity of the human SAMHD1 protein implicated in the Aicardi-Goutires syndrome and HIV-1 restriction. J Biol Chem. 2013;288:8101–8110. 23364794 10.1074/jbc.M112.431148 PMC3605629

[4] Ryoo J, Choi J, Oh C, et al The ribonuclease activity of SAMHD1 is required for HIV-1 restriction. Nat Med [Internet]. 2014;20:936–941. Available from: http://www.nature.com/doifinder/10.1038/nm.3626 10.1038/nm.3626 PMC4318684 25038827

[5] Seamon KJ, Sun Z, Shlyakhtenko LS, et al SAMHD1 is a single-stranded nucleic acid binding protein with no active site-associated nuclease activity. Nucleic Acids Res. 2015;43:6486–6499. 26101257 10.1093/nar/gkv633 PMC4513882

[6] Lahouassa H, Daddacha W, Hofmann H, et al SAMHD1 restricts the replication of human immunodeficiency virus type 1 by depleting the intracellular pool of deoxynucleoside triphosphates. Nat Immunol [Internet]. 2012;13:223–228. Available from: http://www.nature.com/doifinder/10.1038/ni.2236 10.1038/ni.2236 PMC3771401 22327569

[7] Rice GI, Bond J, Asipu A, et al Mutations involved in Aicardi-Goutières syndrome implicate SAMHD1 as regulator of the innate immune response. Nature Genetics [Internet]. 2009;41:829–832. Available from: http://www.nature.com/doifinder/10.1038/ng.373 10.1038/ng.373 PMC4154505 19525956

[8] Rehwinkel J, Maelfait J, Bridgeman A, et al SAMHD1-dependent retroviral control and escape in mice. EMBO J [Internet]. 2013;32:2454–2462. Available from: http://emboj.embopress.org/cgi/doi/10.1038/emboj.2013.163 10.1038/emboj.2013.163 PMC3770946 23872947

[9] Clifford R, Louis T, Robbe P, et al SAMHD1 is mutated recurrently in chronic lymphocytic leukemia and is involved in response to DNA damage. Blood. 2014;123:1021–1031. 24335234 10.1182/blood-2013-04-490847 PMC3924925

[10] Franzolin E, Pontarin G, Rampazzo C, et al The deoxynucleotide triphosphohydrolase SAMHD1 is a major regulator of DNA precursor pools in mammalian cells. Proceedings of the National Academy of Sciences [Internet]; 2013; 110:14272–14277. Available from: http://www.pnas.org/cgi/doi/10.1073/pnas.1312033110 10.1073/pnas.1312033110 PMC3761606 23858451

[11] Chabes AL, Pfleger CM, Kirschner MW, et al Mouse ribonucleotide reductase R2 protein: a new target for anaphase-promoting complex-Cdh1-mediated proteolysis. Proceedings of the National Academy of Sciences of the United States of America [Internet]; 2003; 100:3925–3929. Available from: http://www.ncbi.nlm.nih.gov/pubmed/12655059%5Cnhttp://www.pubmedcentral.nih.gov/articlerender.fcgi?artid=PMC153024 10.1073/pnas.0330774100 PMC153024 12655059

[12] D’Angiolella V, Donato V, Forrester FM, et al Cyclin F-mediated degradation of ribonucleotide reductase M2 controls genome integrity and DNA repair. Cell [Internet]. 2012;149:1023–1034. Available from. 10.1016/j.cell.2012.03.043 PMC3616325 22632967

[13] Pontarin G, Ferraro P, Bee L, et al Mammalian ribonucleotide reductase subunit p53R2 is required for mitochondrial DNA replication and DNA repair in quiescent cells. Proceedings of the National Academy of Sciences [Internet]; 2012; 109:13302–13307. Available from: http://www.pnas.org/cgi/doi/10.1073/pnas.1211289109 10.1073/pnas.1211289109 PMC3421225 22847445

[14] Cribier A, Descours B, Valadao ALC, et al Phosphorylation of SAMHD1 by Cyclin A2/CDK1 regulates its restriction activity toward HIV-1. Cell Reports. 2013;3:1036–1043. 23602554 10.1016/j.celrep.2013.03.017

[15] White TE, Brandariz-Nunez A, Valle-Casuso JC, et al The retroviral restriction ability of SAMHD1, but not its deoxynucleotide triphosphohydrolase activity, is regulated by phosphorylation. Cell Host and Microbe. 2013;13:441–451. 23601106 10.1016/j.chom.2013.03.005 PMC3864637

[16] St. Gelais C, de Silva S, Hach JC, et al Identification of cellular proteins interacting with the retroviral restriction factor SAMHD1. J Virol [Internet]. 2014;88:5834–5844. Available from: http://jvi.asm.org/cgi/doi/10.1128/JVI.00155-14 10.1128/JVI.00155-14 PMC4019113 24623419

[17] Tang C, Ji X, Wu L, et al Impaired dNTPase activity of SAMHD1 by phosphomimetic mutation of Thr-592. J Biol Chem. 2015;290:26352–26359. 26294762 10.1074/jbc.M115.677435 PMC4646291

[18] Yan J, Hao C, DeLucia M, et al CyclinA2-Cyclin-dependent kinase regulates SAMHD1 protein phosphohydrolase domain. J Biol Chem. 2015;290:13279–13292. 25847232 10.1074/jbc.M115.646588 PMC4505580

[19] Arnold LH, Groom HCT, Kunzelmann S, et al Phospho-dependent Regulation of SAMHD1 oligomerisation couples catalysis and restriction. PLoS Pathogens. 2015;11:1–30. 10.1371/journal.ppat.1005194 PMC4592219 26431200

[20] Bhattacharya A, Wang Z, White T, et al Effects of T592 phosphomimetic mutations on tetramer stability and dNTPase activity of SAMHD1 can not explain the retroviral restriction defect. Scientific Reports [Internet]. 2016;6:31353 Available from: http://www.nature.com/articles/srep31353 10.1038/srep31353 PMC4980677 27511536

[21] Welbourn S, Dutta SM, Semmes OJ, et al Restriction of virus infection but not catalytic dNTPase activity is regulated by phosphorylation of SAMHD1. J Virol [Internet]. 2013;87:11516–11524. Available from: http://jvi.asm.org/cgi/doi/10.1128/JVI.01642-13 10.1128/JVI.01642-13 PMC3807338 23966382

[22] Welbourn S, Strebel K. Low dNTP levels are necessary but may not be sufficient for lentiviral restriction by SAMHD1. Virology [Internet]. 2016;488:271–277. Available from. 10.1016/j.virol.2015.11.022 PMC4744553 26655245

[23] Wittmann S, Behrendt R, Eissmann K, et al Phosphorylation of murine SAMHD1 regulates its antiretroviral activity. Retrovirology [Internet]. 2015;12:103 Available from: http://www.retrovirology.com/content/12/1/103 10.1186/s12977-015-0229-6 PMC4678485 26667483

[24] Bianchi V, Spychala J Mammalian 5′-Nucleotidases. J Biol Chem. 2003;278:46195–46198. 12947102 10.1074/jbc.R300032200

[25] Kara N, Hossain M, Prasanth SG, et al Orc1 binding to mitotic chromosomes precedes spatial patterning during G _1_ phase and assembly of the origin recognition complex in human cells. J Biol Chem [Internet]. 2015;290:12355–12369. Available from: http://www.jbc.org/lookup/doi/10.1074/jbc.M114.625012 10.1074/jbc.M114.625012 PMC4424365 25784553

[26] Rogers S, McCloy R, Watkins DN, et al Mechanisms regulating phosphatase specificity and the removal of individual phosphorylation sites during mitotic exit. BioEssays. 2016;38:S24–32. 27417119 10.1002/bies.201670905

[27] Rogers S, McCloy RA, Parker BL, et al Dataset from the global phosphoproteomic mapping of early mitotic exit in human cells. Data in Brief. 2015;5:45–52. 26425664 10.1016/j.dib.2015.08.010 PMC4564385

[28] Vassilev LT, Tovar C, Chen S, et al Selective small-molecule inhibitor reveals critical mitotic functions of human CDK1. Proceedings of the National Academy of Sciences [Internet]; 2006; 103:10660–10665. Available from: http://www.pnas.org/cgi/doi/10.1073/pnas.0600447103 10.1073/pnas.0600447103 PMC1502288 16818887

[29] Hendzel MJ, Wei Y, Mancini MA, et al Mitosis-specific phosphorylation of histone H3 initiates primarily within pericentromeric heterochromatin during G2 and spreads in an ordered fashion coincident with mitotic chromosome condensation. Chromosoma. 1997;106:348–360. 9362543 10.1007/s004120050256

[30] Chen N-P, Uddin B, Voit R, et al Human phosphatase CDC14A is recruited to the cell leading edge to regulate cell migration and adhesion. Proc Natl Acad Sci [Internet]. 2016;113:990–995. Available from: http://www.pnas.org/lookup/doi/10.1073/pnas.1515605113 10.1073/pnas.1515605113 PMC4743822 26747605

[31] Swingle M, Ni L, Honkanen RE Small-molecule inhibitors of Ser/Thr protein phosphatases: specificity, use and common forms of abuse. Protein Phosphatase Protocols [Internet]. 2009;23–38. Available from: http://link.springer.com/10.1385/1-59745-267-X:23 10.1385/1-59745-267-X:23 PMC2709456 17200551

[32] Takai A, Sasaki K, Nagai H, et al Inhibition of specific binding of okadaic acid to protein phosphatase 2A by microcystin-LR, calyculin-A and tautomycin: method of analysis of interactions of tight-binding ligands with target protein. Bio J [Internet]. 1995;306(Pt 3):657–665. Available from: http://www.pubmedcentral.nih.gov/articlerender.fcgi?artid=1136572&tool=pmcentrez&rendertype=abstract 10.1042/bj3060657 PMC1136572 7702557

[33] Favre B Differential inhibition and posttranslational modification of protein phosphatase 1 and 2A in MCF7 cells treated with calyculin-A, okadaic acid, and Tautomycin. J Biol Chem [Internet]. 1997;272:13856–13863. Available from: http://www.jbc.org/cgi/doi/10.1074/jbc.272.21.13856 10.1074/jbc.272.21.13856 9153244

[34] Herold N, Rudd SG, Ljungblad L, et al Targeting SAMHD1 with the Vpx protein to improve cytarabine therapy for hematological malignancies. Nat Med [Internet]. 2017;23:256–263. Available from: http://www.nature.com/doifinder/10.1038/nm.4265 10.1038/nm.4265 28067901

[35] Badia R, Pujantell M, Torres-Torronteras J, et al SAMHD1 is active in cycling cells permissive to HIV-1 infection. Antiviral Res. 2017;142:123–135. 28359840 10.1016/j.antiviral.2017.03.019

[36] Fleck O, Vejrup-Hansen R, Watson A, et al Spd1 accumulation causes genome instability independently of ribonucleotide reductase activity but functions to protect the genome when deoxynucleotide pools are elevated. J Sci [Internet]. 2013;126:4985–4994. Available from: http://jcs.biologists.org/cgi/doi/10.1242/jcs.132837 10.1242/jcs.132837 PMC3820243 23986475

[37] Gon S, Napolitano R, Rocha W, et al Increase in dNTP pool size during the DNA damage response plays a key role in spontaneous and induced-mutagenesis in Escherichia coli. Proceedings of the National Academy of Sciences [Internet]; 2011; 108:19311–19316. Available from: http://www.pnas.org/cgi/doi/10.1073/pnas.1113664108 10.1073/pnas.1113664108 PMC3228436 22084087

[38] Weinberg G, Ullman B, Martin DW Mutator phenotypes in mammalian cell mutants with distinct biochemical defects and abnormal deoxyribonucleoside triphosphate pools. Proceedings of the National Academy of Sciences of the United States of America; 1981; 78:2447–2451. 10.1073/pnas.78.4.2447 PMC319363 7017732

[39] Hofer A, Crona M, Logan DT, et al DNA building blocks: keeping control of manufacture. Crit Rev Biochemistry Mol Biol [Internet]. 2012;47:50–63. Available from: http://www.tandfonline.com/doi/full/10.3109/10409238.2011.630372 10.3109/10409238.2011.630372 PMC3267527 22050358

[40] Coverley D, Laman H, Laskey RA Distinct roles for cyclins E and A during DNA replication complex assembly and activation. Nat Biol [Internet]. 2002;4:523–528. Available from: http://www.nature.com/doifinder/10.1038/ncb813 10.1038/ncb813 12080347

[41] Errico A, Deshmukh K, Tanaka Y, et al Identification of substrates for cyclin dependent kinases. Adv Enzym Regul [Internet]. 2010;50:375–399. Available from. 10.1016/j.advenzreg.2009.12.001 20045433

[42] Cundell MJ, Hutter LH, Bastos RN, et al A PP2A-B55 recognition signal controls substrate dephosphorylation kinetics during mitotic exit. J Biol. 2016;214:539–554. 10.1083/jcb.201606033 PMC5004449 27551054

[43] Stillman B Deoxynucleoside triphosphate (dNTP) synthesis and destruction regulate the replication of both cell and virus genomes. Proceedings of the National Academy of Sciences [Internet]; 2013; 110:14120–14121. Available from: http://www.pnas.org/cgi/doi/10.1073/pnas.1312901110 10.1073/pnas.1312901110 PMC3761580 23946423

[44] Gelais CS, Kim SH, Ding L, et al A putative cyclin-binding motif in human SAMHD1 contributes to protein phosphorylation, localization, and stability. J Biol Chem. 2016;291:26332–26342. 27815502 10.1074/jbc.M116.753947 PMC5159495

[45] Ferraro P, Franzolin E, Pontarin G, et al Quantitation of cellular deoxynucleoside triphosphates. Nucleic Acids Res. 2009;38:1–7. 20008099 10.1093/nar/gkp1141 PMC2847218

[46] Bianchi V, Borella S, Rampazzo C, et al Cell cycle-dependent metabolism of pyrimidine deoxynucleoside triphosphates in CEM cells. J Biol Chem. 1997;272:16118–16124. 9195907 10.1074/jbc.272.26.16118

[47] Hossain M, Stillman B Opposing roles for DNA replication initiator proteins ORC1 and CDC6 in control of Cyclin E gene transcription. eLife. 2016;5:1–22. 10.7554/eLife.12785 PMC4987141 27458800

[48] Hossain M, Stillman B Meier-Gorlin syndrome mutations disrupt an Orc1 CDK inhibitory domain and cause centrosome reduplication. Genes Dev. 2012;26:1797–1810. 22855792 10.1101/gad.197178.112 PMC3426759

[49] Rampazzo C, Mazzon C, Reichard P, et al 5ʹ-Nucleotidases: specific assays for five different enzymes in cell extracts. Biochem Biophys Res Commun. 2002;293:258–263. 12054593 10.1016/S0006-291X(02)00206-1

