# Supplementary material for: The dNTP triphosphohydrolase activity of SAMHD1 persists during S-phase when the enzyme is phosphorylated at T592
Source: Cell Cycle. 2018 Jul 24;17(9):1102–14. doi: 10.1080/15384101.2018.1480216 (PMC6110608; doi:10.1080/15384101.2018.1480216)
Supplement: Supplemental Material [file kccy-17-09-1480216-s001.pdf]

SUPPLEMENTAL FIGURE 1

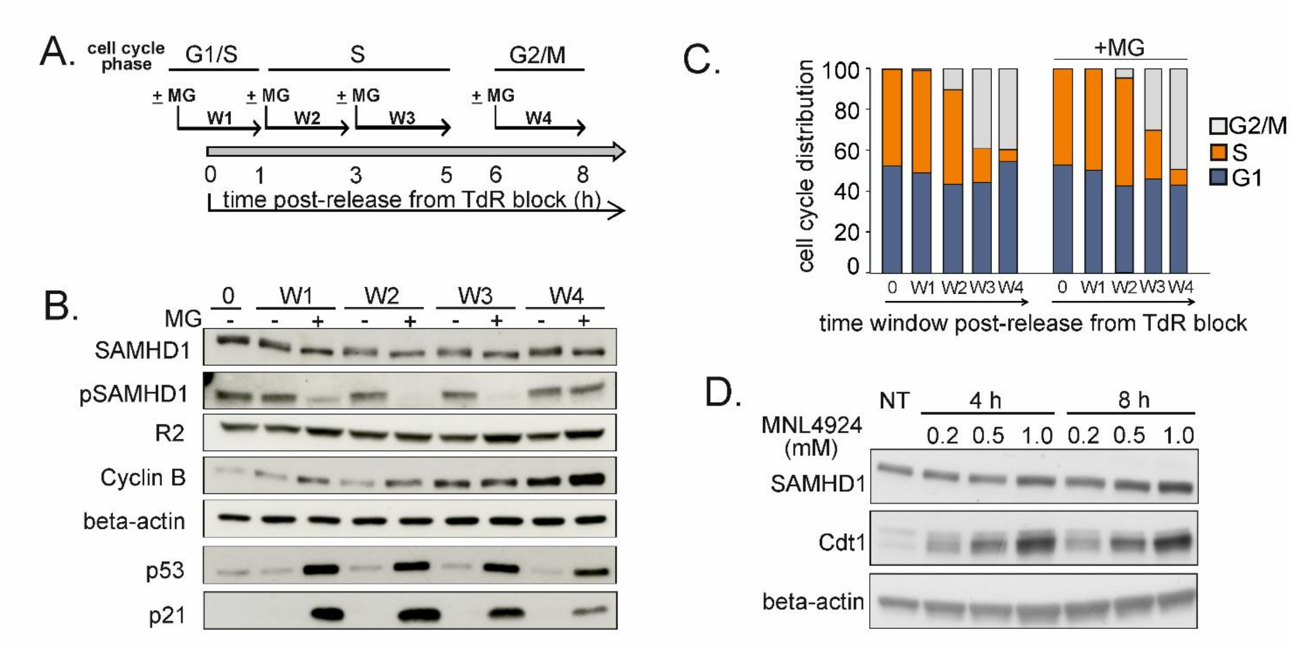

Supplemental Figure 1. Treatment of normal human fibroblasts with inhibitors of protein degradation. A-C. The proteasome inhibitor MG132 inhibits SAMHD1 phosphorylation during S-phase in normal p53-proficient fibroblasts. Normal fibroblasts were synchronized at the G1/S border by double thymidine block. During the release cells were treated with (+) or without (-) MG132 in 2h time windows (W) **(A)**. Samples were collected at the indicated time points for immunoblotting **(B)** and cell cycle analysis **(C)**. The total amount of SAMHD1 (SAMHD1) was detected using an antibody which recognizes both the phosphorylated and non-phosphorylated form, while phosphorylated SAMHD1 (pSAMHD1) was visualized with an antibody against phospho T592. The S-phase induced R2 subunit of RNR, and cyclin B were used as markers of cell cycle progression and beta-actin as a loading control. P53 and p21 were used to evaluate the arrest of cell cycle progression. **D. Inhibition of cullin-RING ubiquitin ligase with MNL4924 does not affect the total amount of SAMHD1.** Asynchronous normal fibroblasts were treated with MNL4924 at the indicated doses and times and samples were collected for immunoblotting. Cdt1 was used as positive control for MNL4924 treatment. SAMHD1 was detected with the antibody recognizing both the phosphorylated and non-phosphorylated form of the protein. Beta-actin: loading control.

SUPPLEMENTAL FIGURE 2

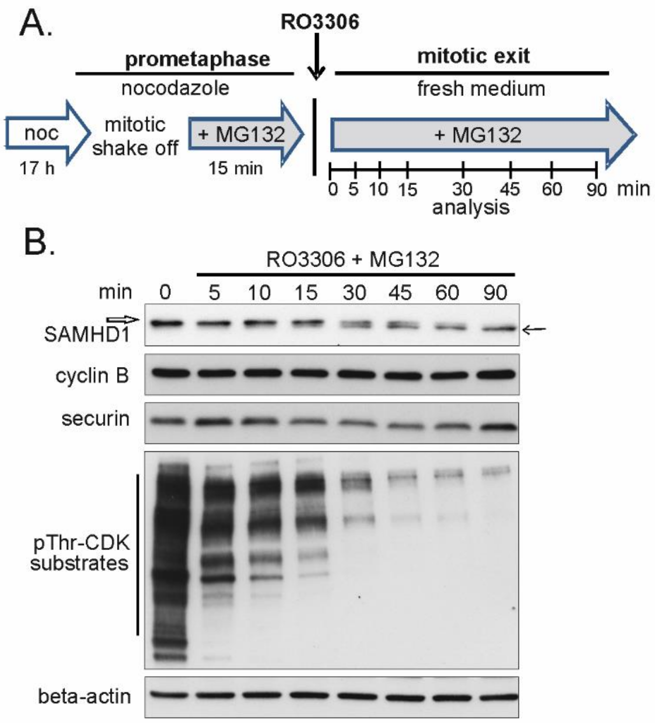

Supplemental Figure 2. SAMHD1 dephosphorylation during mitotic exit in tumor U2OS cells. A. Schematic diagram of the protocol used to synchronize mitotic exit. Samples were collected in prometaphase and at the indicated time points during mitotic exit after RO3306 addition. The proteasome inhibitor MG132 was present 15 min before RO3306 addition and during mitotic exit. **B.** U2OS cells were synchronized as in A. and whole cell extracts were immunoblotted for cyclin B, securin, pThr-CDK substrates and beta-actin (loading control). In the same experiment SAMHD1 phosphorylation was analyzed using an antibody recognizing both the phosphorylated (open arrow) and the non-phosphorylated (black arrow) forms as indicated by a band shift.
